# Workplace Safety Concerns among Co-workers of Responder Returning from Ebola-Affected Country

**DOI:** 10.3201/eid2111.150780

**Published:** 2015-11

**Authors:** Benjamin P. Chan, Elizabeth R. Daly, Elizabeth A. Talbot

**Affiliations:** New Hampshire Department of Health and Human Services, Concord, New Hampshire, USA (B.P. Chan, E.R. Daly, E.A. Talbot);; Geisel School of Medicine at Dartmouth, Lebanon, New Hampshire, USA (E.A. Talbot)

**Keywords:** Ebola, risk perception, viruses, West Africa

## Abstract

We surveyed public health co-workers regarding attitudes toward a physician who returned to New Hampshire after volunteering in the West African Ebola outbreak. An unexpectedly large (18.0%) proportion of staff expressed discomfort with the Ebola responder returning to work. Employers should take proactive steps to address employee fears and concerns.

The largest *Zaire ebolavirus* epidemic reported began in West Africa in December 2013 (*1*) and spread to at least 10 countries, resulting in >27,000 suspected, probable, or confirmed cases of Ebola virus disease (EVD) as of June 2015 (*2*). Arrival of the first case of EVD in the United States resulted in intense public fear and concern, exacerbated by continuous news coverage and misinformation (*3*). A New Hampshire physician, who worked at the New Hampshire Division of Public Health Services (DPHS), volunteered in West Africa; she provided EVD case management training for health care workers in Ebola treatment centers in Sierra Leone but did not provide direct patient care. When she returned to New Hampshire, in accordance with guidelines of the Centers for Disease Control and Prevention and DPHS, she was considered low risk for EVD development and instructed to self-monitor with daily phone checks from DPHS; home quarantine and movement restrictions were not required (*4*,*5*). Given the heightened fear surrounding Ebola, we conducted an assessment of attitudes and intended practices in presumably EVD-educated state public health employees in relation to their Ebola responder co-worker.

## The Study

We developed a Web-based questionnaire (Technical Appendix) and distributed it through workplace email prior to the Ebola responder’s return. The questionnaire provided background information about the returning Ebola responder and asked 26 questions related to 13 hypothetical scenarios and 5 demographic questions. Respondents were asked to predict their reaction, depending on whether the Ebola responder had direct contact with Ebola patients while using appropriate personal protective equipment. 

We grouped responses into dichotomous categories of “comfortable” and “uncomfortable” on the basis of the reported level of comfort or anticipated actions to avoid contact; data were analyzed with SAS v. 9.3 (SAS Institute Inc., Cary, NC, USA). We performed a univariate analysis using the Mantel-Haenszel χ^2^ and McNemar tests to evaluate response comparisons for demographic groups and paired response proportions, respectively. Two-tailed Fisher exact p values were used to assess statistical significance with p<0.05 considered significant. We performed multivariate analyses using logistic regression to assess for associations between the multiple demographic variables and reported comfort level; odds ratios (ORs) were evaluated and considered significant if p<0.05.

A total of 178 (71.2%) of 250 staff members completed the questionnaire. Respondent characteristics are shown in Table 1; scenarios are listed in the first column of Table 2.

**Table 1:** Respondent characteristics for survey assessing workplace comfort levels with co-worker travel to an Ebola-affected country

Characteristic	No. (%) respondents, n = 178*
Sex	
F	133 (78.7)
M	34 (20.1)
Other	2 (1.2)
Age, y; range 23–68, median 51	
20–49	67 (40.6)
50–69	98 (59.4)
Education level	
High school	15 (8.9)
Some college	19 (11.2)
Bachelor’s degree	60 (35.5)
Graduate degree	75 (44.4)
Program area	
Infectious disease	38 (22.8)
Other	129 (77.2)
Clinician	26 (15.1)
*Nonresponders for each demographic question were excluded from the respective proportion calculations. Reponses were missing for program area (n = 11), sex (n = 9), age (n = 13), education level (n = 9), and clinical background (n = 6).	

**Table 2:** Respondent comfort level with co-worker travel to an Ebola-affected country and co-worker contact with Ebola patients

Scenario	No. paired responses, n = 178	No. (%) respondents		% Change (ratio)	p value*
		Travel with contact with Ebola patients	Travel with no contact with Ebola patients		
Uncomfortable with					
Co-worker return to work	178	76 (42.7)	32 (18.0)	--24.7 (2.4)	<0.001
Walking in same hallway as co-worker	177	57 (32.2)	26 (14.7)	−17.5 (2.2)	<0.001
Being in same room as co-worker for meeting	176	65 (36.7)	30 (17.1)	−19.6 (2.1)	<0.001
Sitting in chair next to co-worker	177	88 (49.7)	63 (35.6)	−14.1 (1.4)	<0.001
Standing in line next to co-worker	177	78 (44.1)	47 (26.6)	−17.5 (1.7)	<0.001
Using same restroom as co-worker	176	77 (43.8)	40 (22.7)	−21.1 (1.9)	<0.001
Shaking hands with co-worker	176	85 (48.3)	52 (29.6)	−18.7 (1.6)	<0.001
Hugging co-worker	175	87 (49.7)	59 (33.7)	−16.0 (1.5)	<0.001
Riding in co-worker's car	177	81 (45.5)	51 (28.8)	−16.7 (1.6)	<0.001
Assisting co-worker if he/she fainted	177	95 (53.7)	62 (35.0)	−18.7 (1.5)	<0.001
Eating homemade food made by co-worker	176	95 (54.0)	67 (37.9)	−16.1 (1.4)	<0.001
Would consider not coming to work if co-worker returned	178	37 (20.8)	14 (7.9)	−12.9 (2.6)	<0.001
Would not attend party at co-worker’s home	176	125 (70.6)	82 (46.9)	−23.7 (1.5)	<0.001
*McNemar’s test for paired proportions.					

Even when the Ebola responder had had no contact with an EVD patient, 18.0% of respondents were uncomfortable with the person returning to the workplace; 7.9% reported that they might not come to work if the Ebola responder was present. The proportion of respondents indicating discomfort generally increased in scenarios that described closer and/or more prolonged contact, from walking in the same hallway (14.7%) to attending a holiday party at the Ebola responder’s home (46.9%). When the Ebola responder had direct contact with an EVD patient, the proportion of respondents uncomfortable with each scenario was significantly higher (Table 2), and the percentage of persons who were uncomfortable with a particular scenario increased by an average of 18.3%.

In a univariate analysis, staff members who reported being uncomfortable with the return of an Ebola responder co-worker who had had no contact to EVD patients were 11 times more likely to work in noninfectious disease program areas (OR = 10.7, p = 0.003). No staff person working in the infectious disease program reported discomfort with the Ebola responder returning to work, despite the fact that these employees would have the most contact with the Ebola responder. Because of this strong association, we conducted a subgroup analysis of personnel working outside the infectious disease program, which showed that staff who reported being uncomfortable around the Ebola responder were 3 times more likely to have education below a bachelor’s degree (OR = 2.7, p = 0.039). In univariate or multivariate analyses, no other respondent characteristics were significantly associated with comfort or discomfort.

## Conclusions

Without a doubt, Ebola virus is transmitted through direct contact with infectious body fluids from a symptomatic person (*6*–*10*), but this study suggests that even health department staff may not fully understand this concept. A substantial number of public health staff—with presumably excellent access to accurate EVD information—were uncomfortable with having an asymptomatic Ebola responder return to the workplace. Almost 15% of surveyed staff reported discomfort even walking in the same hallway as an Ebola responder who had had no contact with any EVD patients. Discomfort increased to 35.6% in scenarios describing closer proximity to or physical contact with the returning Ebola responder. Duration of contact also appeared to be a critical factor in perceived risk; more staff reported being uncomfortable sitting in a chair next to the Ebola responder (35.6%) than with shaking the person’s hand (29.6%). This finding may reflect a residual, albeit incorrect, concern over airborne transmission.

Our survey did find that infectious disease staff demonstrated less discomfort. We surmise that these persons had better knowledge of EVD than noninfectious disease staff because they were directly involved in EVD response activities. When we excluded infectious disease staff, having an education level below a bachelor’s degree was the only characteristic significantly associated with increased discomfort. These results are consistent with findings of several public polls: up to two thirds of persons believed that EVD spreads “easily” by multiple routes of transmission, with more than a third concerned that they or a family member could be exposed and get sick from Ebola virus; these beliefs were more common among those with less education (*11*–*13*).

The fear and concern expressed by public health staff are not unique to the United States. Fear and stigmatization in West Africa EVD-epidemic countries, fueled by lack of information and deeply engrained misperceptions, have hindered efforts to control the epidemic and have led to survivor discrimination. Likewise, although state policy allowed this Ebola responder to return to the workplace, the concern expressed by staff created an environment in which she felt she could not work, and she opted to telework. During this period, we conducted outreach to staff through staff meeting presentations, a small group question-and-answer session, and individual meetings to allow persons to ask questions and express concerns. When the monitoring period was over, the Ebola responder returned to work without incident.

Our survey has several limitations. First, scenarios cannot assess the source of discomfort evoked by interacting with a returning traveler; baseline discomfort from a handshake is predictably less than is assisting a co-worker who fainted, regardless of EVD risk. In addition, some responses appear inconsistent. Although riding in a car with an Ebola responder and sitting in an adjacent chair are not different in terms of proximity and contact, the responses differed substantially. Finally, the survey may have drawn attention to the Ebola responder and created concern that would not naturally have occurred if the Ebola responder had returned unannounced to the workplace.

Travelers returning from countries with widespread Ebola virus transmission can elicit strong reactions within their communities. Employers should consider taking active steps to address fears and concerns because workplace reactions and discrimination may have substantial effects on returning Ebola responders. Staff education remains the best approach to alleviating concerns and maintaining a functional workplace, while facilitating the needed humanitarian response to the historic EVD disaster.

Technical Appendix Web-based questionnaire created to assess attitudes and intended practices of state public health employees (presumably educated regarding Ebola virus disease) in relation to a co-worker who was returning from responding to the Ebola outbreak in West Africa.
