# Supplementary material for: Workplace Safety Concerns among Co-workers of Responder Returning from Ebola-Affected Country
Source: Emerg Infect Dis. 2015 Nov;21(11):2077–9. doi: 10.3201/eid2111.150780 (PMC4622258; doi:10.3201/eid2111.150780)
Supplement: Technical Appendix — Web-based questionnaire created to assess attitudes and intended practices of state public health employees (presumably educated regarding Ebola virus disease) in relation to a co-worker who was returning from responding to the Ebola outbreak in West Africa. [file 15-0780-Techapp-s1.pdf]

Article DOI: http://dx.doi.org/10.3201/eid2111.150780

Workplace Safety Concerns among Co-workers of Responder Returning from Ebola-Affected Country

Technical Appendix

The following pages contain the Web-based questionnaire created to assess attitudes and intended practices of state public health employees (presumably educated regarding Ebola virus disease) in relation to a co-worker who was returning from responding to the Ebola outbreak in West Africa. The questionnaire provided background information about the returning Ebola responder and asked 26 questions related to 13 hypothetical scenarios and 5 demographic questions. Respondents were asked to predict their reaction, depending on whether the Ebola responder had direct contact with Ebola patients while using appropriate personal protective equipment.

DPHS Ebola Co-worker
Return Survey

A co-worker of yours at the NH Division of Public Health Services (DPHS) will be going to Liberia to help with Ebola outbreak control measures. When s/he returns, s/he will be monitored by DPHS every day for 21 days so that if Ebola symptoms develop, s/he can immediately be isolated and tested. If no symptoms develop within 21 days of his/her return, then s/he is no longer at risk for developing Ebola and will no longer require public health monitoring.

We are administering this survey to assess your comfort level with your co-worker returning to work and any reservations you may have about interacting with him/her while s/he is being monitored for symptoms of Ebola. This survey is voluntary and confidential and is meant to help us assess workplace attitudes and concerns. The survey has 18 questions and takes approximately 10 minutes to complete.

If you have any questions about this survey, you can contact Beth Daly at 271-4927 or erdaly@dhhs.state.nh.us.

This survey is open until Friday, December 5, 2014. Thank you!

Online Survey Software Powered by novisurvey.net

DPHS Ebola Co-worker
Return Survey

The following questions ask you about your comfort level with various interactions with a co-worker who has possibly been exposed to Ebola virus. Please pay attention to the two proposed scenarios in the heading of each question.

IMPORTANT: All questions refer to interactions that might occur in the 21 days after your coworker returns while he/she is being monitored for Ebola symptoms and NOT after the 21 day monitoring period ends.

1. **How comfortable would you be with your co-worker returning to work?**

	Scenario	
	Your response if the co-worker only traveled to West Africa and had no contact with Ebola patients	Your response if the co-worker had contact with Ebola patients in West Africa while wearing protective equipment
Very comfortable	☐	☐
Mostly comfortable	☐	☐
Mildly uncomfortable	☐	☐
Very uncomfortable	☐	☐

2. **If your co-worker returned to work, would you still come to work as usual?**

	Scenario	
	Your response if the co-worker only traveled to West Africa and had no contact with Ebola patients	Your response if the co-worker had contact with Ebola patients in West Africa while wearing protective equipment
Yes	☐	☐
No	☐	☐
Unsure	☐	☐

3. **If your co-worker was walking down the hallway towards you, what would you do?**

	Scenario	
	Your response if the co-worker only traveled to West Africa and had no contact with Ebola patients	Your response if the co-worker had contact with Ebola patients in West Africa while wearing protective equipment
Continue walking down the hallway and do nothing different than usual	☐	☐
Continue walking down the hallway but try to stay at least a few feet away from him/her	☐	☐
Turn around or go into a room to avoid him/her	☐	☐

4. **How comfortable would you be if you were in the same room with your co-worker during a meeting or conversation?**

	Scenario	
	Your response if the co-worker only traveled to West Africa and had no contact with Ebola patients	Your response if the co-worker had contact with Ebola patients in West Africa while wearing protective equipment
Very comfortable	☐	☐
Mostly comfortable	☐	☐
Mildly uncomfortable	☐	☐
Very uncomfortable	☐	☐
I would not be in the same room with him/her	☐	☐

5. If you were required to be in a meeting with your co-worker around a table, which chair would you pick to sit in?

	Scenario	
	Your response if the co-worker only traveled to West Africa and had no contact with Ebola patients	Your response if the co-worker had contact with Ebola patients in West Africa while wearing protective equipment
Any available seat including the one next to him/her	☐	☐
At least one seat away from him/her	☐	☐
As far away as I can get from him/her	☐	☐
I would not attend the meeting	☐	☐

6. If you were waiting in the cafeteria line for lunch and you realized your co-worker was standing immediately in-front of you or behind you, what would you do?

	Scenario	
	Your response if the co-worker only traveled to West Africa and had no contact with Ebola patients	Your response if the co-worker had contact with Ebola patients in West Africa while wearing protective equipment
Stay in line and do nothing different than usual	☐	☐
Stay in line but try to stay at least a few feet away from him/her	☐	☐
Move to the back of the line	☐	☐
Leave and come back later	☐	☐

7. How comfortable would you be using the same restroom as your co-worker?

	Scenario	
	Your response if the co-worker only traveled	Your response if the co-worker had contact with

	to West Africa and had no contact with Ebola patients	Ebola patients in West Africa while wearing protective equipment
Very comfortable	☐	☐
Mostly comfortable	☐	☐
Mildly uncomfortable	☐	☐
Very uncomfortable	☐	☐
I would not use the same restroom	☐	☐

8. **How comfortable would you be shaking hands with your co-worker (assuming you would normally shake hands)?**

	Scenario	
	Your response if the co-worker only traveled to West Africa and had no contact with Ebola patients	Your response if the co-worker had contact with Ebola patients in West Africa while wearing protective equipment
Very comfortable	☐	☐
Mostly comfortable	☐	☐
Mildly uncomfortable	☐	☐
Very uncomfortable	☐	☐
I would not shake hands	☐	☐

9. **How comfortable would you be hugging your co-worker (assuming you would normally hug him/her)?**

	Scenario	
	Your response if the co-worker only traveled to West Africa and had no contact with Ebola patients	Your response if the co-worker had contact with Ebola patients in West Africa while wearing protective equipment
Very comfortable	☐	☐
Mostly comfortable	☐	☐
Mildly uncomfortable	☐	☐
Very uncomfortable	☐	☐
I would not hug him/her	☐	☐

10. **How comfortable would you be carpooling with your co-worker in him/her car to an off-site meeting?**

	Scenario	
	Your response if the co-worker only traveled to West Africa and had no contact	Your response if the co-worker had contact with Ebola patients in West Africa while wearing

	with Ebola patients	protective equipment
Very comfortable	☐	☐
Mostly comfortable	☐	☐
Mildly uncomfortable	☐	☐
Very uncomfortable	☐	☐
I would not ride in the same car with him/her	☐	☐

11. **If you were with your co-worker at a meeting, and he/she fainted, how comfortable would you be assisting him/her?**

	**Scenario**	
	**Your response if the co-worker only traveled to West Africa and <u>had no contact</u> with Ebola patients**	**Your response if the co-worker <u>had contact</u> with Ebola patients in West Africa while wearing protective equipment**
**Very comfortable**	☐	☐
**Mostly comfortable**	☐	☐
**Mildly uncomfortable**	☐	☐
**Very uncomfortable**	☐	☐
**I would not assist him/her**	☐	☐

12. **How comfortable would you be eating homemade snacks that your co-worker made and brought to work?**

	**Scenario**	
	**Your response if the co-worker only traveled to West Africa and <u>had no contact</u> with Ebola patients**	**Your response if the co-worker <u>had contact</u> with Ebola patients in West Africa while wearing protective equipment**
**Very comfortable**	☐	☐
**Mostly comfortable**	☐	☐
**Mildly uncomfortable**	☐	☐
**Very uncomfortable**	☐	☐
**I would not eat food that he/she made**	☐	☐

13. **Would you go to a holiday party at your co-worker's house in December if he/she was still within 21 days of travel from Liberia and was being actively monitored?**

	Scenario	
	Your response if the co-worker only traveled to West Africa and had no contact with Ebola patients	Your response if the co-worker had contact with Ebola patients in West Africa while wearing protective equipment
**Yes**	☐	☐
**No**	☐	☐
**Unsure**	☐	☐

Online Survey Software Powered by novisurvey.net

DPHS Ebola Co-worker
Return Survey

1. How old are you

Age (in years) ☐

2. What is your sex?

- ○ Female
- ○ Male
- ○ Other

3. Are you a clinician (nurse, physician, etc)?

- ○ Yes
- ○ No
- ○ Unsure

4. What Bureau do you work in?

- ○ Infectious Disease Control
- ○ Public Health Laboratories
- ○ Other Bureau or not assigned to a Bureau

5. What is your highest attained educational level?

- ○ Less than high school graduate
- ○ High school diploma
- ○ Trade school or an associate degree program
- ○ Bachelor degree (BA, BS, etc.)
- ○ Master or Doctoral degree (MA, MS, PhD, MD, etc.)

Online Survey Software Powered by novisurvey.net
